# Postnatal Acetaminophen and Potential Risk of Autism Spectrum Disorder among Males

**DOI:** 10.3390/bs10010026

**Published:** 2020-01-01

**Authors:** Seth S. Bittker, Kathleen R. Bell

**Affiliations:** 1 Ronin Institute, Montclair, NJ 07043, USA; 2 Ontario College of Teachers, Toronto, ON M5S 0A1, Canada

**Keywords:** ASD, paracetamol, APAP, ibuprofen, epidemiology, analgesic

## Abstract

Some evidence from the literature suggests that postnatal acetaminophen exposure may be associated with increased risk of autism spectrum disorder (ASD). Using a data set obtained from a previous study that was derived from an Internet-based survey among parents on 1515 children from the US, an adjusted odds ratio (aOR) and gender-specific aORs for doses of postnatal acetaminophen provided before age two were calculated against the outcome of ASD. Separately, parental uncertainty on the number of doses of acetaminophen provided was analyzed. A population attributable fraction (PAF) associated with postnatal acetaminophen exposure before age two for ASD among males was also estimated. Postnatal acetaminophen exposure, measured in doses before age two, was found to be associated with ASD among male children (aOR 1.023, CI 1.005–1.043, *p* = 0.020*), and parental uncertainty on the number of doses of acetaminophen provided before age two was also found to be associated with ASD. Using this data set, the PAF associated with postnatal acetaminophen was estimated to be about 40% of the risk of ASD among male children in the US. These results suggest the possibility that postnatal acetaminophen may be a significant contributor to the risk of ASD among males in the US.

## 1. Introduction

An earlier iteration of this paper was presented as a poster session at the International Society for Autism Research Annual Meeting in Montreal, Canada, in May 2019 [1].

Autism spectrum disorder (ASD) is a neurodevelopmental disorder characterized by restricted and repetitive behaviors and deficits in social communication [2]. Two prospective case-control studies have found associations between prenatal maternal acetaminophen exposure and types of ASD [3,4]. Two separate retrospective case-control studies, one of which was conducted by the authors of this article, found that postnatal acetaminophen exposure is associated with increased risk of ASD [5,6].

Additional epidemiological evidence points to a possible role for acetaminophen exposure in the induction of some cases of ASD. For example, Becker and Schultz observed that the highly publicized product tampering cases of cyanide-laced Tylenol brand acetaminophen, which led to significant decreases in acetaminophen sales in 1982 and 1986, coincided with plateaus in the incidence of ASD in California [7]. Separately, a study using Danish medical records found that circumcision in infancy is associated with ASD [8]. The authors of this Danish study hypothesized that this association may be due to the pain of the procedure increasing the risk of ASD [8]. Yet this seems unlikely, as circumcision without analgesia was common throughout history until recent decades [9]. As analgesia for infant circumcision has become more common, acetaminophen has often been used to relieve the pain of the procedure [10]. Thus, an alternative explanation for the association observed between circumcision and ASD is that acetaminophen provided coincident or following the procedure may increase the risk of ASD. Others have previously made this same observation [11,12].

Animal studies suggest a connection between postnatal acetaminophen and the development of neurological conditions. In mice, exposure to large doses of acetaminophen early in life has been found to impair behavior and cognitive function in later life [13,14]. In rats, exposure to large doses of acetaminophen early in life has been found to degrade neurotransmission, motor function, spatial memory, and social behavior in later life [15,16]. In humans, ASD is often associated with poor motor function [17], deficits in spatial working memory [18], and deficits in social behavior [19]. Thus, exposure to large doses of acetaminophen in rodents early in life has lifelong effects that appear to be consistent with some of the characteristics of ASD in humans.

As the authors have observed previously, studies on biochemistry suggest that in general children with ASD metabolize acetaminophen less efficiently than unaffected children [5]. Specifically, in children with ASD, the levels of free sulfate [20,21], reduced glutathione [20,21,22,23], cysteine [20,21,22,23], and S-adenosyl methionine [20,21,22,23] in plasma are significantly lower than in controls. Animal studies have found that decreased availability of each of these same compounds is associated with inefficient metabolism of acetaminophen, and as a result, increased risk of acetaminophen toxicity [24,25,26,27,28]. Indeed, a case-control study of acetaminophen metabolism in children with ASD and unaffected controls found abnormalities in acetaminophen metabolism in those with ASD that suggest those with ASD in aggregate metabolize acetaminophen less efficiently than controls [29].

Some genetic research supports this empirical finding. First, two variants of the *SLC13A1* gene, which regulates sulfate transport, are relatively common in ASD [30], and mouse models suggest that these same variants decrease the efficiency of acetaminophen metabolism and increase the propensity for acetaminophen toxicity [31]. Second, genetic variants that decrease the efficiency of the *CTH* gene increase the risk of ASD [32], and mouse models suggest that such variants increase susceptibility to acetaminophen toxicity [33].

These biochemical and genetic findings suggest that children with ASD in aggregate have greater susceptibility to acetaminophen toxicity than unaffected children. While acetaminophen toxicity is usually viewed as a hepatic issue rather than a neurological one, experiments in rats have found that acetaminophen is toxic to neurons at concentrations below those required to induce acute liver failure [34]. In this light, the above suggests that those with ASD may be at increased risk of neuronal damage from significant acetaminophen exposure. It seems plausible that such neuronal damage may increase the risk of ASD in those who would otherwise be unaffected or exacerbate the pathology of ASD in those who are already affected.

Yet, many practitioners continue to recommend acetaminophen for treating fever in infancy [35], for treating teething pain in infancy [36], and for prophylactic relief of pain during inoculation [37]. Relatedly, acetaminophen is the most widely used over-the-counter medication in infancy and early childhood in the US [38].

It is unclear how the widespread use of acetaminophen in infancy and early childhood is appropriate given the evidence highlighted above. Plausibly, its widespread use may be justified if the association between acetaminophen and ASD that has been observed in human studies is not causative. This possibility seems unlikely to the authors since the studies highlighted above show that acetaminophen has significant negative behavioral and cognitive effects in rodents, that acetaminophen is neurotoxic to rodents, and that the biochemistry and genetics of those with ASD in aggregate suggest that those with ASD may be especially susceptible to the effects of acetaminophen due to inefficient metabolism.

Alternatively, even if postnatal acetaminophen is a causative risk factor for ASD, its continued widespread use in infancy and early childhood could be justified if the contribution to the overall risk of ASD from postnatal acetaminophen is extremely low. Thus, some quantification of the possible risk of ASD in the population in aggregate associated with postnatal acetaminophen exposure has great relevance. This study was initiated to attempt to quantify this potential risk.

Studies on risk factors for disease often use odds ratios (ORs) as measures of association [39]. However, the magnitude of an OR is a function of the units in which it is measured [40], and an OR does not account for the prevalence of the exposure in the population [41]. Thus, an OR alone will not provide a useful estimate of aggregate risk of a disease due to a particular factor across the population [41]. Population attributable fraction (PAF) is an alternative measure [42]. It represents the proportion of the overall risk of a disease in a population that is associated with a particular factor [42]. Thus, while calculations of ORs are included in this study, an estimate of PAF associated with postnatal acetaminophen is fundamental to it.

## 2. Methods

### 2.1. Data Set

The underlying data for this study was obtained from an Internet-based survey of parents living in the US that was conducted in the Spring of 2017. Survey participants were recruited through Facebook groups, listservs, websites, and the friends of participants. Participants were also recruited with the assistance of the Interactive Autism Network Research Database, a partnership of the Kennedy Krieger Institute and the Simons Foundation.

This same data set was described in a previous article [5]. Parent participants who had a child with ASD were asked about their youngest child with ASD. Parent participants who did not have a child with ASD were asked about their youngest child between ages 3 to 12. The case set was defined from the former set by further restricting it to consist of only children ages 3 through 12 who had an ASD diagnosis by a professional and who were not known by their parents to have a genetic condition of high penetrance for ASD. The control set was defined from the latter set by further restricting it to consist of children ages 3 through 12 who did not have ASD or a related condition. After exclusions, the sample included 1001 cases and 514 controls.

The survey consisted of between 22 and 25 questions depending upon whether the child in question had ASD. It included one question on the number of doses of acetaminophen consumed by the child before his or her second birthday and another question on the number of doses of ibuprofen consumed by the child before his or her second birthday.

### 2.2. Construction of Variables

A set of dose-dependent categorical variables was constructed to represent the discrete answers to the acetaminophen and ibuprofen questions from the survey. Two separate scaled exposure variables for acetaminophen and ibuprofen were constructed by associating each answer for each range of doses with the lower bound of that range. For example, the answers to the question on the number of doses of acetaminophen provided before age two were: “None”, “1–3”, “4–15”, “16–64”, “64+” and “I’m not sure”. These answers were associated with numbers: 0, 1, 4, 16, 64, and “not available” (NA).

Demographic variables were also constructed. Some were scaled variables, such as age of the child or education, and others were categorical variables, such as Midwest, which indicated whether the respondent lives in the Midwest. The eight demographic variables considered in this study are: gender, age of the child, ethnicity, Midwest, South, maternal education, age of mother at birth of child, and relationship to the child.

### 2.3. Statistical Analysis

Analyses were run using the R statistical package. The threshold for statistical significance on *p*-values in this study was set to be 0.05. Crude dose-dependent Wald ORs and 95% confidence intervals (CIs) were computed for the acetaminophen and ibuprofen categorical variables against the outcome of ASD.

Logistic regressions were run to compute ORs and adjusted odds ratios (aORs) for scaled variables acetaminophen, ibuprofen, and the two jointly. Analogous calculations were run for males-only and females-only subsets. Categorical aORs were also computed for each acetaminophen dose stratum.

A consistent set of demographic covariates was used for all logistic regressions with covariates against the outcome of ASD using the following procedure. For each of the two scaled analgesic variables in the full data set, a preliminary logistic regression was run where the candidate set of covariates consisted of the eight demographic variables against the outcome of ASD. Variable selection in these preliminary logistic regressions was made using the Akaike information criterion (AIC). AIC is a measure of relative model quality [43]. The union of all covariates included in each of these two logistic regressions was used as the set of demographic covariates to be included in all logistic regressions with covariates against the outcome of ASD.

Tests for interaction between each of the scaled analgesic variables and gender against the outcome of ASD were also conducted using logistic regressions with interaction terms.

To determine if any variables are associated with the parent not knowing how many doses of acetaminophen the child consumed, logistic regressions were run to compute aORs for the outcome of “NA for acetaminophen”. Potential covariates were the eight demographic variables and ASD status. Variable selection was determined objectively using AIC. The same procedure was followed to obtain aORs for the outcome of “NA for ibuprofen”.

Percentages of male cases exposed to each discrete dose stratum of postnatal acetaminophen were obtained directly from the data set. The PAF among males was calculated for each acetaminophen dose stratum using a standard formula for PAF for variables with exposure levels as provided by Rockhill and derived by Walter and Kleinbaum [42,44,45]:

(1) $$\frac{p_{i}\;\left(RR_{i}-1\right)}{1+\;\sum_{j=0}^{k}p_{j}\;\left(RR_{j}-1\right)}$$

This formula provides the PAF for stratum *i* where there are k strata in total, *p_j_* is the proportion of the case population exposed to stratum *j*, and *RR_j_* is the risk ratio between cases and controls for stratum *j*.

Using the rare disease assumption as defined by Greenland and Thomas [46], the risk ratio for each dose stratum was approximated by the categorical aOR for that dose stratum. The aggregate PAF associated with postnatal acetaminophen was then obtained by summing the per stratum PAF across the acetaminophen strata.

## 3. Results

### 3.1. Demographics

Table 1 provides the demographic characteristics of the participants. It is an abridged and edited version of an analogous table from the authors’ previous article that relied on this data set [5]. To summarize, case children were 1.8 years older than controls and were somewhat more ethnically diverse than controls. Relatively more case children lived in the South and relatively less were from the Midwest than controls. Case mothers were 1.2 years younger than control mothers and somewhat less well-educated in aggregate than control mothers.

### 3.2. Crude Dose-Dependent Wald ORs

Table 2 provides aggregate, males-only, and females-only crude dose-dependent Wald ORs for acetaminophen and ibuprofen against the outcome of ASD. Two of these dose-dependent variables have statistically significant associations: 64+ doses of acetaminophen and 64+ doses of acetaminophen in males.

### 3.3. Gender-Specific ORs and aORs

Table 3 provides aggregate, males-only, and females-only ORs and aORs for the scaled variables acetaminophen and ibuprofen against the outcome of ASD. Based on the procedure highlighted in the methods section, using AIC, all eight demographic variables were included as covariates in the aORs in the full data set—they are: gender, age of the child, ethnicity, Midwest, South, maternal education, age of mother at birth of child, and relationship. These same variables with the exception of gender were used as covariates in the gender-specific models.

With respect to gender-specific interaction effects, the *p*-value of an acetaminophen X gender variable in a regression with acetaminophen, gender and covariates is 0.253, and the *p*-value of an ibuprofen X gender variable in an analogous regression with ibuprofen, gender, and covariates is 0.723.

Table 4 provides statistics on a joint model including both acetaminophen and ibuprofen and males-only and females-only versions of this same model.

### 3.4. aORs for NA for Acetaminophen and NA for Ibuprofen

Table 5 provides aORs for the demographic variables and ASD regressed against the outcomes of “NA for acetaminophen” and “NA for ibuprofen” using AIC as the criteria for variable inclusion.

### 3.5. PAF for ASD Associated with Acetaminophen among Males

Table 6 provides statistics on each acetaminophen dose stratum, aORs for acetaminophen for each stratum, and the calculated PAF per stratum. Summing the PAFs across strata, the PAF for ASD associated with postnatal acetaminophen among males is estimated to be 43.7%.

## 4. Discussion

### 4.1. Demographics

The demographic differences between the case and control sets are generally explainable based on the construction of the survey and ASD epidemiology. For example, control parents were asked about their youngest child between 3 and 12 years old, while case parents were asked about their youngest child with ASD. It seems certain that many of the case parents, who answered as instructed regarding their youngest child with ASD, actually had a younger unaffected child between 3 and 12 years old. Thus, by construction, one would expect the average age of case children to be older than the average age of control children. In addition, ASD is often diagnosed late [47]. This would also suggest an older age for case children in this study, since some of the younger control children might later be diagnosed with ASD. Thus, it is not surprising that cases were on average 1.8 years older than controls.

### 4.2. Units

While most of the aORs in this study for the scaled variables may seem small in magnitude, the magnitude of ORs and aORs are dependent on the units of the underlying variables [40]. In this study, the scaled acetaminophen and ibuprofen variables are measured in units of doses provided before age two. If instead these variables were expressed as dichotomous variables relative to a threshold number of doses, the magnitude of the aORs would be much larger.

### 4.3. Possible Recall Bias

In some retrospective studies on severe disease, case respondents may overestimate the subject’s exposure to variables that they believe could be factors in inducing the disease [48]. While such recall bias does not always occur, in cases where it does occur, it can tilt results toward an association between the exposure in question and the disease [48,49,50].

An important question is whether recall bias played a significant role in the results of this study. It is notable that among the two analgesic scaled exposure variables in this study, the associations with acetaminophen are much stronger than with ibuprofen. Thus, if recall bias did significantly affect the results for the analgesic variables, its role seems to have been significantly greater for the acetaminophen variable than for the ibuprofen variable. Also, recall bias did not appear to affect results on a vitamin D drop variable and a prenatal folate variable that were collected simultaneously [5]. Specifically, as the authors noted in their previous study with this data set, the survey responses suggested that oral vitamin D drop exposure is weakly associated with ASD after adjusting for covariates, and prenatal folate has no association with ASD but the aOR is in the direction of increasing risk [5]. If recall bias was fundamental to these results, the expected directions of association with vitamin D drops and folate would be in the opposite direction of what was observed.

Also, in the present study, a statistically significant association was found between the “I’m not sure” response on the acetaminophen question and ASD. It seems unlikely that such a disproportionate “I’m not sure” response among cases would be a result of recall bias, as the usual pattern when recall bias does occur is that case participants recall exposures that did not occur or over-estimate exposures that did occur [48].

### 4.4. Gender-Specific ORs and aORs

Table 2 highlights that 64+ doses of acetaminophen before age two has a statistically significant association with ASD. The OR is greater in magnitude for the analogous variable in the males-only set. While the results in this table also suggest the possibility of associations with acetaminophen at more modest doses and potential associations with ibuprofen, none of the other results from this table are statistically significant.

The *p*-values from Table 3 show that the association of the males-only acetaminophen scaled variable with ASD is statistically significant, while the females-only acetaminophen scaled variable is far from statistically significant. Similarly, the aOR for the males-only acetaminophen variable is much greater in magnitude than the analogous females-only variable. As there are more males than females in this data set, one might be tempted to attribute the much weaker association among females to the greater number of males in the sample.

However, the *p*-values and aORs for acetaminophen in the males-only set and the full data set suggest the possibility that the association between acetaminophen and ASD is somewhat stronger in the males-only set than in the full set, despite the fact that the males-only set is significantly smaller. In addition, the acetaminophen results contrast with the ibuprofen results, where in general, the associations are much weaker and are not characterized by a stronger association among males than in the full set.

While the *p*-value for the interaction term with acetaminophen is much lower than with ibuprofen, neither one is statistically significant. One possibility is that there is no interaction effect with gender for either of these variables. Another possibility is that there is an interaction effect, but the sample was insufficiently sized to show it based on a *p*-value threshold of 0.05. This seems plausible for acetaminophen, as sample sizes required to accurately measure interaction effects are much larger than sample sizes needed for measuring primary effects [51].

While Table 3 hints at a possible weak association between ibuprofen and ASD, the much larger *p*-values for ibuprofen in the joint models from Table 4 indicate that any association between ibuprofen and ASD could conceivably be a result of ibuprofen use being correlated with acetaminophen use.

### 4.5. Potential Confounders

A natural question is whether other variables that were not examined in this study could explain the relatively strong association observed between postnatal acetaminophen and ASD among males. One possibility is that cases may have greater indications of use for acetaminophen than controls. For example, it is plausible that cases, who are those who later receive an ASD diagnosis, could have greater susceptibility to fevers and hence be more likely to receive acetaminophen than controls.

If this scenario is driving the association between acetaminophen and ASD observed in this study, then one would expect a similarly strong association between ibuprofen and ASD, as both are used as antipyretics. Yet, the associations between ibuprofen and ASD observed in this study are much weaker than those between acetaminophen and ASD, and this is especially true among males.

Another potential cofounder is prenatal maternal acetaminophen exposure. As highlighted in the Introduction, prenatal maternal acetaminophen exposure has been found to be associated with ASD [3,4]. There is also evidence that maternal use of acetaminophen tends to be correlated with use in school children [52]. Thus, it seems plausible that prenatal acetaminophen exposure is a correlated variable that could be causative and confounding.

However, other factors in addition to maternal use may be involved in parental proclivity to provide over-the-counter medications to their children [53,54]. In addition, the associations between prenatal acetaminophen and ASD from the literature seem to be relatively weak, despite being obtained from large data sets [3,4]. Thus, it seems unlikely that a correlation with prenatal acetaminophen, a variable with relatively weak association with ASD, would fully explain the strong association observed between postnatal acetaminophen among males and ASD observed in the present study with a smaller data set.

### 4.6. NA for Acetaminophen and NA for Ibuprofen

Table 5 shows that demographic factors alone do not account for the pattern of NA responses on the acetaminophen and ibuprofen questions observed in this study. Specifically, AIC results in the inclusion of ASD status as a statistically significant variable in regression models for the outcome of NA responses. This suggests that the pattern of NA responses for these variables can be seen as additional confirmation of an association between analgesic use and ASD status.

Recall that NA responses came from respondents who selected “I’m not sure”. For example, on the acetaminophen question, the parent respondents who selected this answer would have been those who were uncertain about how many doses of acetaminophen were consumed by their child. It seems probable to the authors that a parent would have remembered if acetaminophen was never provided to her child or if it was only provided rarely. Thus, the NA responses on the acetaminophen question may be indicative of higher than average levels of acetaminophen exposure. As ASD is statistically significantly associated with NA responses on the acetaminophen question from Table 5, calculations which exclude NA responses like those in Table 3 may underestimate the strength of the association between acetaminophen and ASD. The same is true of the association between ibuprofen and ASD.

### 4.7. PAF for ASD Associated with Acetaminophen among Males

There is considerable imprecision around the estimate of PAF for ASD associated with postnatal acetaminophen exposure among males. As noted above, the risk ratios were approximated by the aORs for the dose strata in the PAF calculations. Greenland and Thomas found that this approximation is only likely to induce significant bias in cases where the prevalence of the disease in the population in question is greater than 10% [46]. As ASD prevalence among eight-year-old males is about 2.7% per Baio et al. [55], this approximation is unlikely to induce significant bias in the PAF calculation.

While this approximation is unlikely to introduce significant bias, the aORs on the acetaminophen per dose strata are imprecise, especially for the lower dose strata where the CIs are wide. As these aORs are fundamental to the PAF calculation, this imprecision introduces imprecision into the PAF estimate.

In addition, while the aORs for the dose strata were adjusted for seven covariates, it seems likely that there may be other variables, which are correlated with postnatal acetaminophen exposure and are not included in this analysis, that may contribute to ASD risk. For example, these might include indications of use and prenatal acetaminophen. As previously explored, it seems unlikely that either of these variables would fully account for the association between postnatal acetaminophen and ASD observed in this study, but either of these variables could account for a portion of this association. In a scenario where such correlated variables contribute to risk, the PAF could be overestimated [56].

Conversely, it is possible that the aOR for acetaminophen among males is underestimated due to the disproportionate “I’m not sure” responses among case participants. As explored above, these responses may have resulted in relative underestimation of higher dose exposure among case children. This scenario would tend to lead to underestimation of the PAF.

Thus, while the data presented here suggests that there is a non-trivial PAF associated with postnatal acetaminophen among males, there is significant imprecision around this estimate.

The reader may wonder why the authors have not calculated the PAF for postnatal acetaminophen among females. Given that any association between acetaminophen exposure and ASD among females is not statistically significant in this data set, as Table 3 indicates, and relatedly the CIs are as a result very wide, the authors do not believe a meaningful estimate of the PAF for ASD among females can be obtained using this data set.

### 4.8. Strengths and Limitations

Some limitations of this study arose from its reliance on a data set that was based on an Internet survey. For example, parents’ answers to survey questions were not independently verified. In addition, information on the size of the doses provided was not obtained. It also would have been desirable if there were greater demographic similarity between the case and control sets. In addition, the significant number of NA responses on the acetaminophen question increases the uncertainty around the results obtained, even if this uncertainty likely points in the direction of increased risk.

A strength of this study is that it rigorously shows that uncertainty on the number of doses of acetaminophen provided before age two is associated with ASD. Separately, expressing the main result from this study as a PAF offers additional transparency and clarity on its potential relevance.

## 5. Conclusions

This study finds that postnatal acetaminophen measured in doses consumed before age two is associated with ASD risk in male children (aOR 1.023, CI 1.005–1.043, *p* = 0.020 *). If this data set is representative, then the PAF for postnatal acetaminophen is approximately 40% of the risk of ASD among males in the US. This may be an overestimate, as other correlated variables that were not included in this analysis may also contribute to risk of ASD. While it is possible that the association between postnatal acetaminophen and ASD in males is not causative, this study shows that if the association is causative, the contribution to risk in aggregate from this variable may be a substantial portion of the risk of ASD. Prospective studies on this variable are needed.

## Figures and Tables

**Table 1:** Demographics.

Variable	Cases	Controls
*n*	1001	514
Gender of child—male (*n*, %)	801 (80.0)	268 (52.1)
Age of child (mean, SD)	7.3 (2.9)	5.5 (2.6)
Maternal age at birth of child (mean, SD)	30.0 (5.6)	31.2 (4.6)
Ethnicity of child (*n*, %)		
White (non-Hispanic)	773 (77.2)	438 (85.2)
Hispanic/Latino	104 (10.4)	30 (5.8)
African American/Black	39 (3.9)	5 (1.0)
Asian	19 (1.9)	11 (2.1)
Other	66 (6.6)	30 (5.8)
Regions of residence in US (*n*, %)		
Northeast	180 (18.0)	103 (20.0)
Midwest	240 (24.0)	176 (34.2)
South	362 (36.2)	108 (21.0)
West	219 (21.9)	127 (24.7)
Maternal education (*n*, %)		
Grade school or some high school	10 (1.0)	6 (1.2)
High school	80 (8.0)	22 (4.3)
Some college	272 (27.2)	72 (14.0)
Bachelor’s or Associate’s degree	394 (39.4)	208 (40.5)
Graduate or professional degree	244 (24.4)	206 (40.1)
I’m not sure	1 (0.1)	-
Relationship to child (*n*, %)		
Biological mother	978 (97.7)	497 (96.7)
Biological father	21 (2.1)	15 (2.9)
NA	2 (0.2)	2 (0.4)

Abbreviations: SD, standard deviation; %, percentage of cases or controls.

**Table 2:** Dose dependent Wald ORs relative to no exposure.

		Cases	Controls			
Exposure	Dose	*n* (%)	*n* (%)	OR	95% CI	*p*-Value
Acetaminophen	None	54 (5.4)	45 (8.8)	Reference		
	1–3	174 (17.4)	91 (17.7)	1.593	0.996–2.549	0.0511
	4–15	378 (37.8)	216 (42.0)	1.458	0.949–2.241	0.0839
	16–63	191 (19.1)	107 (20.8)	1.488	0.938–2.359	0.0904
	64+	26 (2.6)	4 (0.8)	5.417	1.759–16.68	0.0015 *
	Not sure	178 (17.8)	51 (9.9)	-	-	-
Ibuprofen	None	185 (18.5)	115 (22.4)	Reference		
	1–3	167 (16.7)	91 (17.7)	1.141	0.808–1.612	0.4549
	4–15	304 (30.4)	180 (35.0)	1.050	0.780–1.413	0.7481
	16–63	141 (14.1)	74 (14.4)	1.184	0.822–1.706	0.3634
	64+	17 (1.7)	6 (1.2)	1.761	0.675–4.597	0.2422
	Not sure	187 (18.7)	48 (9.3)	-	-	-
Acetaminophen in males	None	37 (4.6)	20 (7.5)	Reference		
	1–3	134 (16.7)	52 (19.4)	1.393	0.741–2.619	0.3023
	4–15	308 (38.5)	110 (41)	1.514	0.842–2.719	0.1634
	16–63	156 (19.5)	55 (20.5)	1.533	0.821–2.864	0.1782
	64+	21 (2.6)	2 (0.7)	5.676	1.206–26.72	0.0167 *
	Not sure	145 (18.1)	29 (10.8)	-	-	-
Ibuprofen in males	None	147 (18.4)	61 (22.8)	Reference		
	1–3	126 (15.7)	47 (17.5)	1.112	0.740–1.742	0.6415
	4–15	245 (30.6)	83 (31)	1.225	0.830–1.807	0.3060
	16–63	118 (14.7)	44 (16.4)	1.113	0.705–1.758	0.6465
	64+	13 (1.6)	3 (1.1)	1.798	0.495–6.535	0.3668
	Not sure	152 (19)	30 (11.2)	-	-	-
Acetaminophen in females	None	17 (8.5)	25 (10.2)	Reference		
	1–3	40 (20)	39 (15.9)	1.508	0.707–3.218	0.2866
	4–15	70 (35)	106 (43.1)	0.971	0.489–1.929	0.9333
	16–63	35 (17.5)	52 (21.1)	0.990	0.467–2.097	0.9787
	64+	5 (2.5)	2 (0.8)	3.677	0.638–21.19	0.1274
	Not sure	33 (16.5)	22 (8.9)	-	-	-
Ibuprofen in females	None	38 (19)	54 (22)	Reference		
	1–3	41 (20.5)	44 (17.9)	1.324	0.731–2.399	0.3541
	4–15	59 (29.5)	97 (39.4)	0.864	0.511–1.463	0.5871
	16–63	23 (11.5)	30 (12.2)	1.089	0.550–2.158	0.8059
	64+	4 (2)	3 (1.2)	1.895	0.401–8.957	0.4137
	Not sure	35 (17.5)	18 (7.3)	-	-	-

Notes: * for *p* values < 0.05. Abbreviations: OR, Wald odds ratio; 95% CI, 95% Wald confidence interval.

**Table 3:** Gender-specific ORs and aORs for scaled variables.

		Unadjusted			Adjusted for Demographics		
Set	Variable	OR	95% CI	*p*-Value	aOR ^a^	95% CI	*p*-Value
Full set	Acetaminophen (doses)	1.015	1.003–1.028	0.018 *	1.016	1.003–1.032	0.0259 *
	Ibuprofen (doses)	1.008	0.996–1.021	0.210	1.012	0.997–1.027	0.1106
Males-only	Acetaminophen (doses)	1.017	1.001–1.036	0.046 *	1.023	1.005–1.043	0.0200 *
	Ibuprofen (doses)	1.006	0.991–1.024	0.464	1.014	0.997–1.034	0.1356
Females-only	Acetaminophen (doses)	1.009	0.988–1.031	0.401	1.005	0.981–1.030	0.7196
	Ibuprofen (doses)	1.007	0.986–1.029	0.521	1.007	0.982–1.033	0.5737

Notes: ^a^ Covariates for the aORs are: gender, age of the child, ethnicity, Midwest, South, maternal education, age of mother at birth of child, and relationship. * for *p*-values < 0.05. Abbreviations: OR, odds ratio; aOR, adjusted odds ratio; 95% CI, 95% confidence interval.

**Table 4:** Joint models for scaled variables.

Set	*n*	Variable	aOR ^a^	95% CI	*p*-Value
Full set	1257	Acetaminophen (doses)	1.018	0.998–1.039	0.0892
		Ibuprofen (doses)	1.000	0.979–1.020	0.9701
Males-only	871	Acetaminophen (doses)	1.025	1.001–1.053	0.0577
		Ibuprofen (doses)	0.998	0.973–1.024	0.8713
Females-only	386	Acetaminophen (doses)	1.002	0.968–1.041	0.8960
		Ibuprofen (doses)	1.004	0.965–1.040	0.8295

Notes: ^a^ Covariates for the aORs are: gender, age of the child, ethnicity, Midwest, South, maternal education, age of mother at birth of child, and relationship. Abbreviations: aOR, adjusted odds ratio; 95% CI, 95% confidence interval.

**Table 5:** aORs for not available (NA) for acetaminophen and NA for ibuprofen.

Model	*n*	Variable	aOR ^a^	95% CI	*p*-Value
NA for Acetaminophen	1514	Age of Child	1.141	1.085–1.201	<0.0001 *
		ASD	1.466	1.040–2.095	0.0318 *
		Education	0.846	0.724–0.991	0.0372 *
NA for Ibuprofen	1514	Age of Child	1.144	1.088–1.203	<0.0001 *
		Education	0.827	0.708–0.967	0.0171 *
		ASD	1.539	1.074–2.237	0.0210 *
		Gender (male)	1.307	0.931–1.860	0.1289

Notes: ^a^ Covariates for the aORs are: gender, age of the child, ethnicity, Midwest, South, maternal education, age of mother at birth of child, and relationship. * for *p*-values < 0.05. Abbreviations: aOR, adjusted Odds Ratio; 95% CI, 95% confidence interval.

**Table 6:** PAF associated with postnatal acetaminophen among males by strata.

Acetaminophen Doses	Cases *n* (%)	aOR ^a^	95% CI	PAF (%)
None	37 (5.6)	Reference		
1–3	134 (20.4)	1.360	0.683–2.680	4.13
4–15	308 (47)	1.685	0.881–3.165	18.11
16–63	156 (23.8)	1.824	0.909–3.616	11.03
64+	21 (3.2)	6.805	1.595–47.633	10.45

Notes: ^a^ Covariates for the aORs are: age of the child, ethnicity, Midwest, South, maternal education, age of mother at birth of child, and relationship. Abbreviations: aOR, adjusted odds ratio; 95% CI, 95% confidence interval; PAF, population attributable fraction.

## Abbreviations

aOR	Adjusted odds ratio
ASD	Autism spectrum disorder
CI	95% confidence interval
OR	Odds ratio
NA	Not available
PAF	Population attributable fraction
US	United States
